# plantGIR: a genomic database of plants

**DOI:** 10.1093/hr/uhae342

**Published:** 2024-12-05

**Authors:** Zhuo Liu, Chenhao Zhang, Jinghua He, Chunjin Li, Yanhong Fu, Yongfeng Zhou, Rui Cao, Haibin Liu, Xiaoming Song

**Affiliations:** School of Life Sciences/School of Basic Medical Sciences/ Key Laboratory For Quality of Salt Alkali Resistant TCM of Hebei Administration of TCM, North China University of Science and Technology, Tangshan, Hebei 063210, China; School of Life Sciences/School of Basic Medical Sciences/ Key Laboratory For Quality of Salt Alkali Resistant TCM of Hebei Administration of TCM, North China University of Science and Technology, Tangshan, Hebei 063210, China; School of Life Sciences/School of Basic Medical Sciences/ Key Laboratory For Quality of Salt Alkali Resistant TCM of Hebei Administration of TCM, North China University of Science and Technology, Tangshan, Hebei 063210, China; School of Life Sciences/School of Basic Medical Sciences/ Key Laboratory For Quality of Salt Alkali Resistant TCM of Hebei Administration of TCM, North China University of Science and Technology, Tangshan, Hebei 063210, China; School of Life Sciences/School of Basic Medical Sciences/ Key Laboratory For Quality of Salt Alkali Resistant TCM of Hebei Administration of TCM, North China University of Science and Technology, Tangshan, Hebei 063210, China; National Key Laboratory of Tropical Crop Breeding, Shenzhen Branch, Guangdong Laboratory of Lingnan Modern Agriculture, Key Laboratory of Synthetic Biology, Ministry of Agriculture and Rural Affairs, Agricultural Genomics Institute at Shenzhen, Chinese Academy of Agricultural Sciences, Shenzhen 518000, China; National Key Laboratory of Tropical Crop Breeding, Tropical Crops Genetic Resources Institute, Chinese Academy of Tropical Agricultural Sciences, Haikou 571101, China; School of Life Sciences/School of Basic Medical Sciences/ Key Laboratory For Quality of Salt Alkali Resistant TCM of Hebei Administration of TCM, North China University of Science and Technology, Tangshan, Hebei 063210, China; School of Life Sciences/School of Basic Medical Sciences/ Key Laboratory For Quality of Salt Alkali Resistant TCM of Hebei Administration of TCM, North China University of Science and Technology, Tangshan, Hebei 063210, China; School of Life Sciences/School of Basic Medical Sciences/ Key Laboratory For Quality of Salt Alkali Resistant TCM of Hebei Administration of TCM, North China University of Science and Technology, Tangshan, Hebei 063210, China

Dear Editor,

With the advancement of genomic sequencing technology and the continuous reduction in costs, an increasing number of species have undergone whole-genome sequencing, which provides a wealth of resources for comparative genomics and functional genomics research [1–3]. In the field of plant genomics and bioinformatics research, the standardization, uniformity, and accessibility of genomic data are extremely important. However, the diversity of data submission pathways has resulted in numerous data download channels. Consequently, it is challenging to ensure the accuracy and standardized consistency of the genomic data. Furthermore, some databases are no longer accessible for use. Typically, genomic data for individual species are scattered across a multitude of databases, with sequence IDs being prone to change during the transfer process between these repositories. Although numerous databases exist, each focusing on specific families, genera, or taxonomic categories [4–7], there remains a void for a comprehensive repository that includes genomic data for all sequenced plant species.

Here, we have meticulously curated the original download links from 797 publications, amassing a comprehensive dataset from 110 diverse databases, encompassing 1117 plant species. To facilitate the use of these genomic data resources by a wider range of relevant researchers, we have developed the plant genomic information resources (plantGIR, http: //plantgir.cn/) data-sharing platform, which is a treasure trove of genomic insights into these 1117 species from lower to higher plants. The database mainly included three principal genomic file formats, including coding sequences, protein sequences, and general feature format files.

Moreover, we have conducted a thorough identification of genes associated with flowering, auxin, and anthocyanin biosynthesis, along with transcription factors. This dataset deposited in plantGIR serves as a vital foundation for advancing genomic studies and enhancing our understanding of plant genetics. Transcription factor (TF) families were identified using PlantTFDB website (https://planttfdb.gao-lab.org). The flowering, anthocyanin, and auxin genes were identified by BLASTP (*E*-value <1e−5; identity >60%; score > 150) with *Arabidopsis thaliana* according to the previous reports, with manual verification for accuracy [8–10]. Finally, a total of 200 906 flowering, 60 438 auxin, and 42 626 anthocyanin biosynthesis genes were identified in 1117 plant genomes. The plantGIR database contains 45 792 677 gene annotations obtained using the Gene Ontology, nonredundant, Swiss-Prot, and TrEMBL databases. Moreover, 2 206 412 transcription factors were detected in all 1117 plants, which was further divided into 59 gene families. Among these transcription factors, there was the most number for basic helix-loop-helix (bHLH) (191862) gene family, followed by myeloblastosis (MYB) (167682) and ethylene-responsive factor (ERF) (166880) gene families.

On plantGIR databse, we present an intuitive interface for showcasing functional genes and transcription factors in the introduction section on the homepage (Fig. 1A), with clickable links that lead to detailed display pages (Fig. 1C-E). Our advanced fuzzy search enables users to input just the initial part of a term, prompting the database to reveal corresponding species, complete with taxonomic details and a statistic of species within each category (Fig. 1A). By clicking on these numbers, users can comprehensively view of all species within that category, including literature references, while clicking on a species name transports them to an elaborate information display, offering rich data and analytical insights specific to that species (Fig. 1B). We also employ a dynamic word cloud to spotlight the top ten most-accessed species (Fig. 1A), selecting these leads to a data-monitoring hub that illustrates the top 20 species in terms of search and download frequency (Fig. 1K). We display the provenance of species data, detailing the publication year and the original databases obtaining the species information. Additionally, we provide easy access to all associated publication articles, streamlining the user’s quest for in-depth information (Fig. 1L). Our download options are versatile, featuring a table-based interface for swift selection of desired taxonomic levels, with the capability for multi-select and bundled downloads. We also support File Transfer Protocol (FTP downloads), empowering users to remotely access our data directly from their computers. The third option is an interactive tree diagram of our species, where clicking on a species name unveils a trove of information and analytical findings (Fig. 1J).

**Figure 1:**
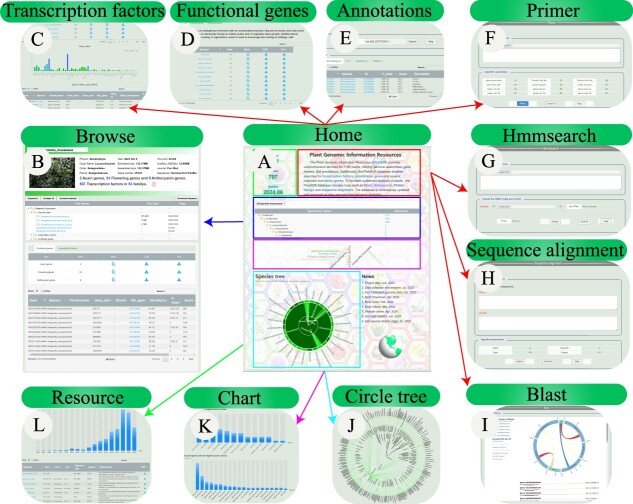
Overview of the main interface and internal features of the plantGIR database. (A) Home interface. (B) Species browse interface. (C) Transcription factors interface. (D) Functional genes interface. (E) Annotations interface. (F-I) Tools interface. (J) Species tree download interface. (K) Visit charts interface. (L) Data resource

Furthermore, we encourage user participation through a data submission portal, inviting contributions of data for species not yet represented in our database, with the assurance that such additions will be promptly featured. To enhance the user experience, we integrate four essential tools for online analysis: Primer Design (Fig. 1F), Hmmsearch (Fig. 1G), Sequence Alignment (Fig. 1,H), and Blast (Fig. 1I), catering to the diverse analytical needs of users.

In contrast to traditional databases, we have enhanced our monitoring capabilities by showing the top 20 species in real time, ranked by page views and download times. To establish a more effective connection with users, we have implemented an email registration and login system. Moreover, with the aim of simplifying the download process, we have provided three distinct download methods. Users can download data directly from our server through FTP links or download one or multiple species concurrently via our website, providing a flexible and user-friendly experience.

The essence of plantGIR is its role as a comprehensive data-sharing platform that integrates a wide array of data from recent sequencing studies, complemented by the incorporation of four widely used bioinformatic tools. This platform is not just a repository, it is a dynamic environment that is constantly evolving. With a forward-looking perspective, plantGIR is committed to the continuous refinement of its datasets and analytical capabilities. This dedication ensures that the platform remains at the forefront of comparative genomics, meeting the growing demands of this field in the era of big data.

Our ambition is to transform plantGIR into a pivotal hub for data aggregation and online analytical services. We anticipate that plantGIR will extend its functionalities to encompass the display of preliminary genomic data analyses, streamlining the user journey to identify and access the information they seek. By fostering a vibrant community centered on data sharing, plantGIR welcomes contributions from researchers with their valuable insights and suggestions for continuous improvement. It is through this spirit of collaboration that we aim to sustain and enhance the platform’s utility, ensuring plantGIR remains a vital resource for the scientific community.

## Data Availability

All materials and data related to this study are available in our plantGIR database (http://plantgir.cn).
